# Physiological and biomechanical comparison of overground, treadmill, and ergometer handrim wheelchair propulsion in able-bodied subjects under standardized conditions

**DOI:** 10.1186/s12984-020-00767-2

**Published:** 2020-10-17

**Authors:** Rick de Klerk, Vera Velhorst, Dirkjan (H.E.J.) Veeger, Lucas H. V. van der Woude, Riemer J. K. Vegter

**Affiliations:** 1 Centre for Human Movement Sciences, University Medical Centre Groningen, University of Groningen, Antonius Deusinglaan 1, 9713 AV Groningen, The Netherlands; 2 Mechanical, Maritime and Materials Engineering, Delft University of Technology, Postbus 5, 2600 AA Delft, The Netherlands; 3 Centre for Rehabilitation, University Medical Centre Groningen, Antonius Deusinglaan 1, 9713 AV Groningen, The Netherlands

**Keywords:** Dynamometry, Biomechanics, Power output, Gross mechanical efficiency

## Abstract

**Background:**

Handrim wheelchair propulsion is often assessed in the laboratory on treadmills (TM) or ergometers (WE), under the assumption that they relate to regular overground (OG) propulsion. However, little is known about the agreement of data obtained from TM, WE, and OG propulsion under standardized conditions. The current study aimed to standardize velocity and power output among these three modalities to consequently compare obtained physiological and biomechanical outcome parameters.

**Methods:**

Seventeen able-bodied participants performed two submaximal practice sessions before taking part in a measurement session consisting of 3 × 4 min of submaximal wheelchair propulsion in each of the different modalities. Power output and speed for TM and WE propulsion were matched with OG propulsion, making them (mechanically) as equal as possible. Physiological data and propulsion kinetics were recorded with a spirometer and a 3D measurement wheel, respectively.

**Results:**

Agreement among conditions was moderate to good for most outcome variables. However, heart rate was significantly higher in OG propulsion than in the TM condition. Push time and contact angle were smaller and fraction of effective force was higher on the WE when compared to OG/TM propulsion. Participants used a larger cycle time and more negative work per cycle in the OG condition. A continuous analysis using statistical parametric mapping showed a lower torque profile in the start of the push phase for TM propulsion versus OG/WE propulsion. Total force was higher during the start of the push phase for the OG conditions when compared to TM/WE propulsion.

**Conclusions:**

Physiological and biomechanical outcomes in general are similar, but possible differences between modalities exist, even after controlling for power output using conventional techniques. Further efforts towards increasing the ecological validity of lab-based equipment is advised and the possible impact of these differences -if at all- in (clinical) practice should be evaluated.

## Background

The repetitive and relatively high loads on the upper-extremities during handrim wheelchair propulsion, associated with an increased risk of pain and pathology [1–3], are a continued concern addressed in wheelchair research [4, 5]. Ideally, research would assess the user during overground testing in the environment they are daily exposed to, as this has the highest ecological validity [5]. During overground propulsion the power output necessary at a certain velocity is dependent on a number of uncontrollable factors such as floor-type, slope, cross-slope and air resistance, besides individual factors of the wheelchair-combination as a whole like weight, frontal area tire-pressure and internal frictional losses. Moreover, there are effects of optical flow, and additional requirements such as braking and cornering [6]. Therefore, experimental conditions overground are difficult to control and it can be challenging to consistently collect enough consecutive push cycles without a sufficiently spacious laboratory environment [7].

Various other options for conducting studies on wheelchair propulsion exist, such as, motorized treadmills or wheelchair ergometers with each having their own advantages and disadvantages [8]. The advantage of these lab-based systems, in general, is the better standardization and the ability to collect multiple subsequent push cycles, increasing data reliability [9]. However, stationary systems offer no visual flow or meaningful context which reduces task complexity and might confound data obtained from these methods. In fact, ergometers mostly remove the need for steering and balancing as task elements in handrim wheelchair propulsion, making it the most abstract measurement modality [6].

Research in gait has shown that, while treadmills are mechanically valid [10], differences between overground and treadmill modalities exist [11–19], while others have argued that differences are minimal [14, 16, 20]. Similar studies for wheelchair propulsion, however, are lower in number and have also yielded mixed results [6, 21–25]. Stephens and Ensberg [25] found that biomechanical outcome variables for overground propulsion and treadmill propulsion were significantly different. Moreover, Chénier et al. found that wheelchair users perceive speed differently on treadmills compared to overground propulsion [23]. However, in different studies Kwarciak et al. [24] and Mason et al. [21] found that physiological and biomechanical parameters in treadmill propulsion highly correlate with overground propulsion at specific treadmill settings. Koontz et al. [22] also found correlations between overground and ergometer wheelchair propulsion kinetics ranging from poor-good depending on the outcome parameter.

A possible explanation of these mixed results could (in part) be the lack of standardization for power output and/or speed in those experiments. As of yet, there are no studies that compared overground, treadmill, and ergometer wheelchair propulsion when power output and speed are matched, even though the required methodology has been well described and adopted in the research literature [5, 26] and is available to most labs [4]. Spatiotemporal variables are known to be dependent on factors influencing power output such as speed and slope [27]. Finally, only qualitative [22] and discrete quantitative [21, 22, 24] comparisons have been made, whereas continuous analysis of handrim biomechanics might also yield useful information as the biomechanical context is immediately apparent [28].

The goal of this study is therefore to compare the physiological, spatiotemporal, and kinetic characteristics of wheelchair propulsion between overground, treadmill, and ergometer handrim wheelchair propulsion while controlling for power output and speed using available standardization methods [26]. Results from this study can be used to better translate research to the field or to improve existing testing protocols.

## Methods

### Participants

A convenience sample of seventeen able-bodied subjects, age(21.6 ± 2.4 years), mass(69.6 ± 8.2 kg), height(1.74 ± 0.07 m), sex(4M/13F), handedness(16R/1L), volunteered in the study. Participants had no previous experience in wheelchair propulsion and did not have any contraindications for exercise (Par-Q, [29]). Written informed consent was obtained before participants were enrolled. The study was approved by the local ethics committee of the Centre for Human Movement Sciences Groningen, University Medical Centre Groningen, The Netherlands.

### Protocol

**Fig. 1:**
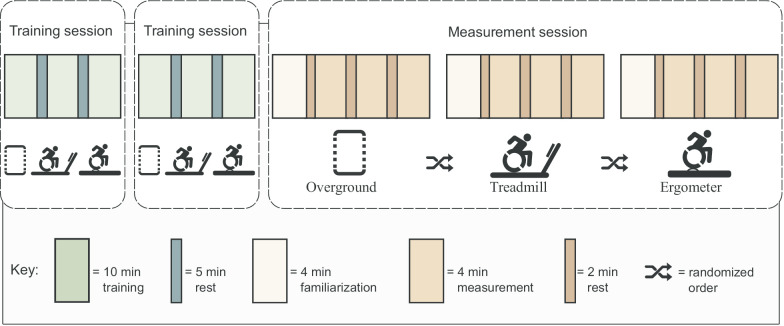
Research design: two training sessions were followed by a longer measurement session. Each session was independent and on a different day. During the measurements, participants performed three blocks of wheelchair propulsion per modality (overground, treadmill, and ergometer) at 1.11 m/s. The last minute of data were used for analysis

The current study employed a within-subjects design with three sessions (two training sessions and one measurement session on separate days) in which participants did a trial of overground, treadmill, and ergometer handrim wheelchair propulsion in a randomized order (Fig. 1). Two training sessions at a fixed speed of 1.11 m/s were performed to familiarize participants with wheelchair propulsion in general [30, 31] and with the specific testing modalities [20]. Before the measurement session, a coast-down test [32] was performed to determine the static rolling resistance overground which was used to standardize power output among conditions. During the measurement session, the first block was used to familiarize the participant with the modality again. Thereafter, three blocks of four minutes of steady state propulsion were performed at 1.11 m/s in each of the three testing modalities.

All tests were performed in the same non-individualized Küschall k-series wheelchair with instrumented wheels. The right wheel was replaced with a force sensing Optipush Biofeedback System (MAX Mobility, USA, [33]) and the left wheel was replaced with an inertial dummy wheel (total weight = 22 kg). Tires were inflated before every session to 6 bars (600 kPa). Oxygen consumption was measured with a mobile breath-by-breath spirometer (K5, Cosmed, Italy). Heart rate was determined with a heart rate monitor (Garmin, USA) connected to the same system.

### Standardization of power output

To ensure all conditions were (at least mechanically) as equal as possible, the power output and speed were standardized for all subjects [26]. The overground condition served as a reference for the treadmill and ergometer condition. Frictional forces during overground propulsion were estimated with a back-and-forth coast-down test on the same surface as the overground condition, using velocity data from the measurement wheel [32]. Friction was assumed to be constant and independent of velocity. Treadmill rolling friction was individually determined with a drag test and matched with the friction obtained from the coast-down tests using a pulley system [5]. Ergometer power output was set in the ergometer software by changing the rolling friction coefficient in the associated software [34] for each individual wheelchair-user combination.

### Overground propulsion

**Fig. 2:**
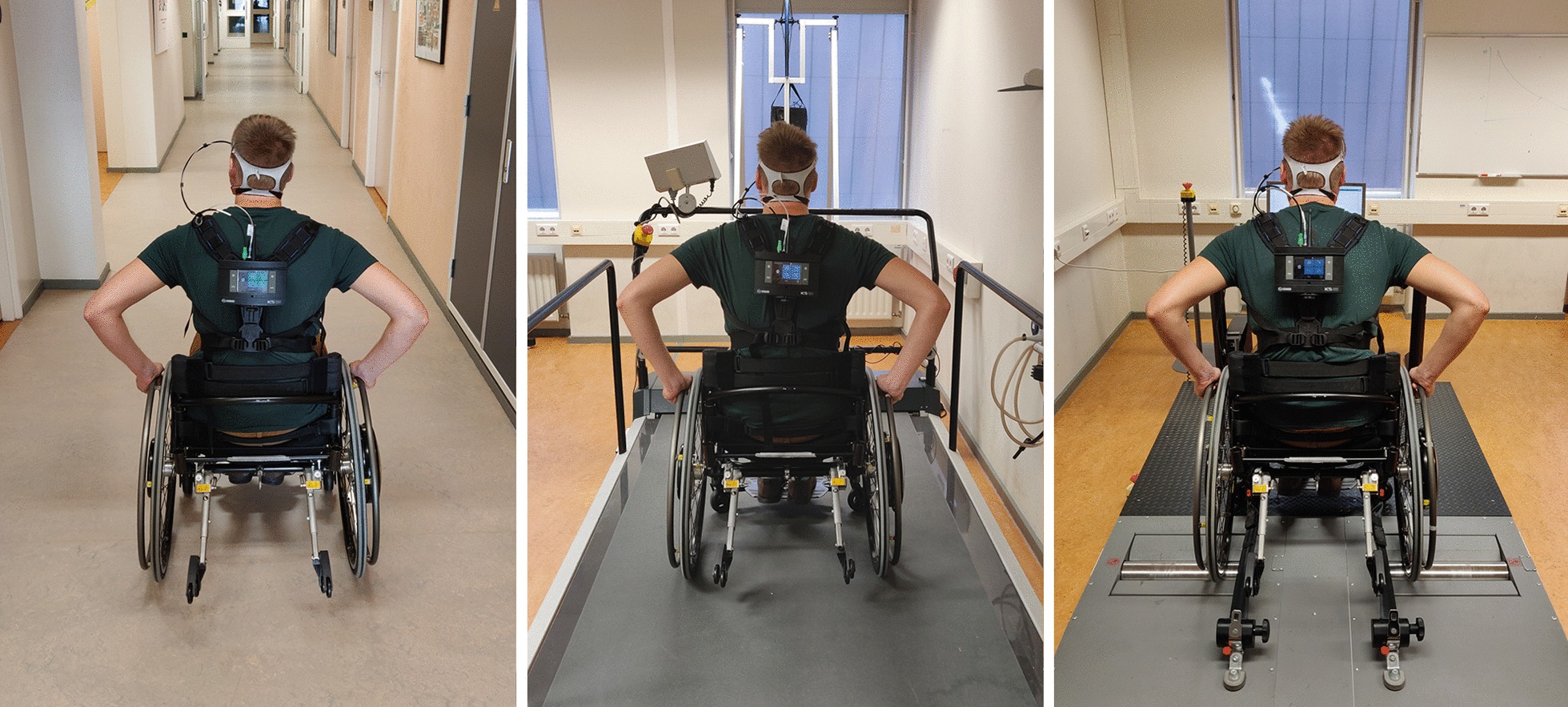
Overview of the three different conditions: overground, treadmill, and ergometer wheelchair propulsion

The participants propelled the wheelchair in an empty rectangular hospital hallway (long straights: 25 m, short straights: 9 m, width: 2.5 m) with a smooth linoleum floor (Fig. 2). All practice and measurement sessions were performed in clockwise laps. The subjects were instructed to propel the wheelchair at a constant speed using feedback from the measurement wheel. They received visual feedback that was updated each push cycle from the measurement wheel on a laptop screen attached to their lap with hook-and-loop fasteners. No adjustments to rolling resistance were made during the overground blocks. A timer was used to mark the long sections of the course as only the straight corridor—and not the corners—were used for analysis.

### Treadmill propulsion

Participants performed the same exercises on a wide wheelchair treadmill (1.20 × 2.00 m, Forcelink, Culemborg, The Netherlands). A safety system that stopped the treadmill if a participant were to roll too far to the back of the treadmill was present as well as one operator in front of the treadmill with access to a safety stop button and one operator at the back of the treadmill. After performing a drag-test [5], a pulley-system was attached to the wheelchair to match the power output requirements of overground propulsion. Participants were instructed to stay in the middle of the treadmill and received no additional (visual) feedback of their speed. Treadmill velocity was verified with a CDT-2000HD calibrated tachometer (Checkline, USA).

### Ergometer propulsion

The ergometer trials were performed on a computer-controlled Lode Esseda wheelchair ergometer (Lode B.V., Groningen, The Netherlands, described in [34]). The wheelchair was secured on the ergometer with four straps. Before each trial a static and dynamic calibration were performed to null the system. The simulated inertia on the ergometer was set to the combined weight of the user and the wheelchair. The simulated friction on the ergometer rollers was set as a constant value based on the coast-down test in the overground condition. Participants received real-time visual feedback of their current speed and heading (speed left, speed right) on a monitor in front of the ergometer. They were instructed to maintain an average speed of 4.0 km/h (1.11 m/s).

### Analysis

Data were collected during each trial of the measurement session and only the last minute was used for analysis, assuming a steady-state had been reached. Physiological, spatiotemporal, and kinetic outcome variables were obtained from the spirometer, and measurement wheel data. Data (pre-) processing and calculations [4, 35], and subsequent statistics were performed using Python 3.7 (Python Software Foundation). Data were first cut so the last minute of each block for each test could be used for analysis. For overground propulsion, only data from the long straights were included. Kinetic data were then filtered using an 4th-order zero-phase Butterworth filter with a 10 Hz cut off frequency and discrete outcomes [4] were calculated (push time, cycle time, contact angle, fraction of effective force, mean torque per push, max torque per push, work per push, and negative work per cycle). Heart rate, energy expenditure, and gross mechanical efficiency were calculated based on the spirometer data.

### Statistical analysis

Agreement among conditions was determined with a two-way random effects, single rater, absolute agreement Intraclass Correlation (ICC(2,1) [36]). ICC values below 0.50 were considered as poor, values between 0.50 and 0.75 as moderate, values between 0.75 and 0.90 as good, and values higher than 0.90 as excellent [37]. Thereafter, a repeated measures analysis of variance (RM-ANOVA) was performed to test for significant differences between modalities. A Friedman test was used if data were not normally distributed. Planned pairwise comparisons were performed after a significant main effect was detected using paired t-tests (or Wilcoxon signed-rank test) without correction to maximize sensitivity, and effect sizes were given. Effect sizes lower than 0.2 were considered small, values between 0.2 and 0.5 as medium, and higher than 0.5 as large [38]. For the continuous analysis, the mean torque and total force of the last twenty push cycles (normalized from 0 to 100% with cubic interpolation) for each participant were compared with paired Statistical Parametric Mapping (SPM) t-tests using the spm1d package [28]. All values are presented as is, without (statistical) correction for power output.

## Results

The results and statistical outcomes are provided in Table 1. The measured power output by the measurement wheel was significantly lower than expected based on the coast-down test for all modalities (p < 0.001), − 16%, − 19%, − 11%, for overground, treadmill, and ergometer propulsion, respectively. Please refer to [26] for a more detailed analysis of external power output. Agreement for power output among conditions was not significant for overground versus treadmill propulsion and moderate for overground versus ergometer and treadmill versus ergometer propulsion. However, there was no significant difference between conditions in power output F(2,32) = 1.614, p = 0.215. There was, however, a small difference in speed (which did not follow a normal distribution) χ^2^(2) = 6.706, p = 0.035 between conditions, specifically for overground/treadmill versus ergometer propulsion (2%). Intraclass correlations for speed were non-significant due to the low variation in results (i.e. their coefficients of variation were lower than 0.02).

**Table 1:** Physiological and biomechanical characteristics for overground (OG), treadmill (TM), and ergometer (WE) wheelchair propulsion (n = 17)

Variable		Mean (± SD)	Contrast	ICC (95% CI)^a^	p-value^b^	p-value	Effect size
*Experimental*							
Power output (W)	OG TM WE	8.12 (1.41) 7.84 (1.92) 8.65 (2.24)	OG vs. TM OG vs. WE TM vs. WE	n.s.^e^ 0.57 (0.15–0.82) 0.52 (0.10–0.79)	0.10 **0.01** **0.00**	n.s.^f^ n.s.^f^ n.s.^f^	
Speed (m/s)	OG TM WE	1.12 (0.02) 1.12 (0.00) 1.14 (0.02)	OG vs. TM OG vs. WE TM vs. WE	n.s.^e^ n.s.^e^ n.s.^e^	0.48 0.57 0.52	0.20^g^ **0.02**^g^ **0.03**^g^	0.7^i^ − 0.6^i^
*Physiological*							
Heart rate (bpm)^h^	OG TM WE	94.49 (11.80) 89.30 (11.31) 92.24 (13.16)	OG vs. TM OG vs. WE TM vs. WE	**0.86** (0.05–0.97) **0.91** (0.73–0.97) **0.88** (0.64–0.96)	**0.00** 0.13 0.07	**0.00**^**c**^ 0.27^**c**^ 0.07^**c**^	− 1.4^d^
Energy expenditure (W)	OG TM WE	208.88 (50.00) 195.97 (40.08) 206.31 (42.47)	OG vs. TM OG vs. WE TM vs. WE	0.74 (0.42–0.90) 0.68 (0.31–0.87) **0.78** (0.49–0.91)	**0.00** **0.00** **0.00**	n.s.^f^ n.s.^f^ n.s.^f^	
Gross mechanical efficiency (%)	OG TM WE	4.07 (1.07) 4.12 (1.10) 4.28 (1.15)	OG vs. TM OG vs. WE TM vs. WE	**0.75** (0.44–0.90) 0.44 (0.00–0.75) 0.55 (0.11–0.81)	**0.00** **0.03** **0.01**	n.s.^f^ n.s.^f^ n.s.^f^	
*Spatiotemporal*							
Push time (s)	OG TM WE	0.35 (0.06) 0.35 (0.07) 0.32 (0.07)	OG vs. TM OG vs. WE TM vs. WE	**0.85** (0.64–0.94) **0.80** (0.34–0.93) **0.83** (0.35–0.95)	**0.00** **0.00** **0.00**	0.96^**c**^ **0.00**^**c**^ **0.00**^**c**^	− 0.8^d^ 0.8^d^
Cycle time (s)	OG TM WE	1.43 (0.47) 1.31 (0.44) 1.28 (0.55)	OG vs. TM OG vs. WE TM vs. WE	**0.85** (0.60–0.95) **0.79** (0.49–0.92) **0.75** (0.44–0.90)	**0.00** **0.00** **0.00**	**0.02**^g^ **0.01**^g^ 0.39^g^	0.6^i^ 0.8^i^
Contact angle (deg)	OG TM WE	71.43 (12.34) 72.28 (13.64) 68.74 (14.01)	OG vs. TM OG vs. WE TM vs. WE	**0.86** (0.66–0.95) **0.87** (0.60–0.95) **0.87** (0.60–0.95)	**0.00** **0.00** **0.00**	0.83^**c**^ **0.00**^**c**^ **0.00**^**c**^	− 1.2^d^ 0.9^d^
*Kinetics*							
Fraction of effective force (%)	OG TM WE	69.28 (10.33) 69.67 (9.86) 73.38 (8.11)	OG vs. TM OG vs. WE TM vs. WE	**0.92** (0.81–0.97) 0.71 (0.29–0.89) 0.69 (0.30–0.88)	**0.00** **0.00** **0.00**	0.69^**c**^ **0.02**^**c**^ **0.03**^**c**^	0.6^d^ − 0.7^d^
Mean torque per push (Nm)	OG TM WE	4.63 (1.06) 4.34 (1.33) 4.65 (1.33)	OG vs. TM OG vs. WE TM vs. WE	**0.76** (0.47–0.91) **0.81** (0.55–0.93) **0.81** (0.55–0.93)	**0.00** **0.00** **0.00**	n.s.^f^ n.s.^f^ n.s.^f^	
Max torque per push (Nm)	OG TM WE	7.83 (2.02) 7.43 (2.41) 8.25 (2.73)	OG vs. TM OG vs. WE TM vs. WE	**0.81** (0.57–0.93) **0.82** (0.57–0.93) **0.77** (0.46–0.91)	**0.00** **0.00** **0.00**	n.s.^f^ n.s.^f^ n.s.^f^	
Work per push (J)	OG TM WE	5.94 (1.94) 5.61 (2.17) 5.64 (2.20)	OG vs. TM OG vs. WE TM vs. WE	**0.82** (0.57–0.93) **0.85** (0.63–0.94) **0.80** (0.53–0.92)	**0.00** **0.00** **0.00**	n.s.^f^ n.s.^f^ n.s.^f^	
Negative work per cycle (J)	OG TM WE	− 1.44 (0.51) − 0.50 (0.20) − 0.42 (0.21)	OG vs. TM OG vs. WE TM vs. WE	n.s.^e^ n.s.^e^ 0.52 (0.10–0.79)	0.23 0.21 **0.01**	**0.00**^g^ **0.00**^g^ 0.07^g^	− 1.0^i^ − 1.0^i^

^a^Two-way random-effects model, single rater, absolute agreement, ≥ good values are bold

^b^p-values for the intraclass-correlations, significant results are bold

^c^Paired-comparison with t-test without correction, significant results are bold

^d^Effect size: Cohen’s d

^e^Non-significant intraclass-correlation

^f^Non-significant main effect

^g^Wilcoxon signed-rank test

^h^n = 14

^i^Rank-biserial correlation

### Physiology

**Fig. 3:**
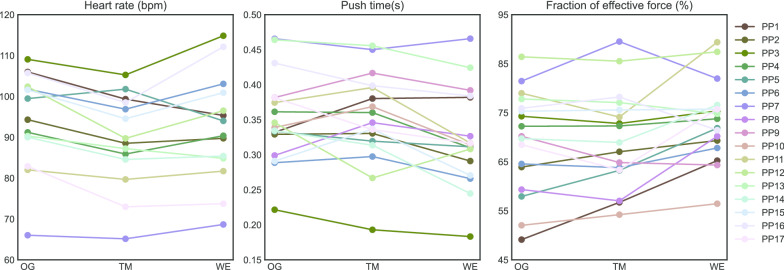
Individual responses on three variables with moderate-excellent agreement but significant differences between modalities

Heart rate showed good agreement among all modalities (Table 1, Fig. 3). A significant main effect of modality was found F(2,26) = 7.998, p = 0.002. Heart rate was significantly different between overground and treadmill propulsion with a large effect size. Energy expenditure showed moderate agreement among overground and treadmill/ergometer propulsion, and good agreement among treadmill and ergometer propulsion. No significant differences were found between the three modalities F(2,32) = 1.526, p = 0.233. Gross mechanical efficiency showed good agreement among overground and treadmill propulsion, but only moderate agreement among overground and ergometer, and poor agreement among treadmill and ergometer propulsion. No significant main effect was found F(2,32) = 0.362, p = 0.699.

### Spatiotemporal outcomes

Agreement for push time was good among all modalities (Table 1, Fig. 3). A significant main effect was found F(2,32) = 7.323, p = 0.002. Participants had a significantly higher push time in the overground/treadmill condition than on the ergometer. Cycle time also showed good agreement among all conditions. Data were not normally distributed and a significant main effect was found χ^2^(2) = 12.118, p = 0.002. Post-hoc comparisons showed that cycle time in the overground condition was higher than in treadmill/ergometer propulsion. Contact angle also showed good agreement among all conditions and no significant main effect was found F(2,32) = 2.547, p = 0.094.

### Kinetics

Fraction of effective force (FEF) showed excellent agreement among overground and treadmill propulsion (Table 1, Fig. 3). However, only moderate agreement was found between overground and ergometer, and treadmill and ergometer propulsion. A significant main effect was found F(2,32) = 5.377, p = 0.010. Post-hoc analysis showed significant differences between overground/treadmill and ergometer propulsion with large effect sizes. Mean and peak torque (T_z_) per push showed good agreement among all conditions and no significant main effects were found F(2,32) = 1.703, p = 0.198, and F(2,32) = 2.628, p = 0.088, respectively. A good agreement among all conditions was found for work per push without a significant main effect χ2(2) = 3.647, p = 0.161. However, negative work per cycle only showed moderate agreement between treadmill and ergometer propulsion. A significant main effect was found χ^2^(2) = 26.941, p = 0.000 and overground propulsion was found to be different from treadmill/ergometer propulsion.

**Fig. 4:**
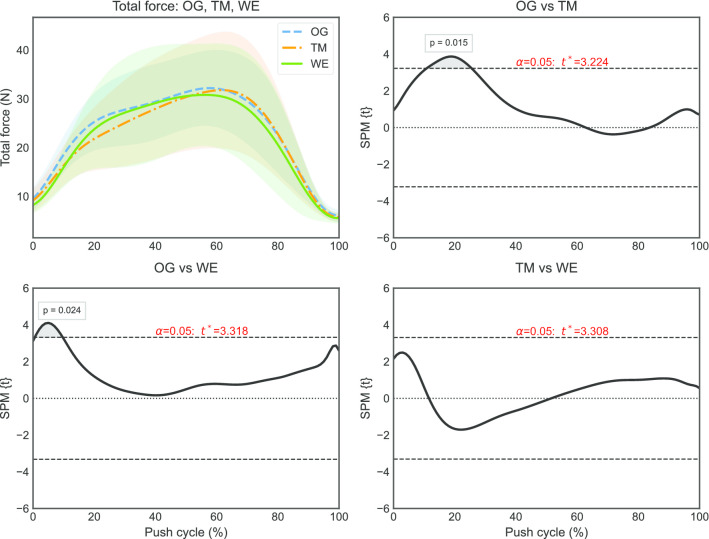
Total force (μ ± σ) and results of SPMs with pointwise t-statistics and p-values (n = 17)

**Fig. 5:**
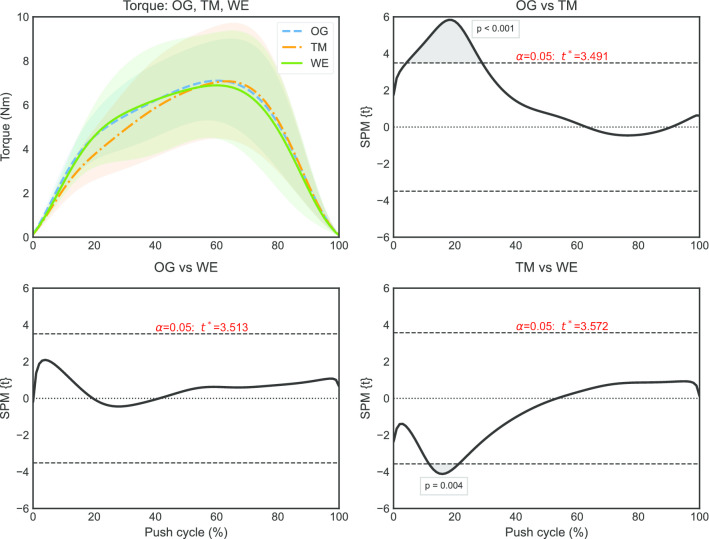
Torque (μ ± σ) and results of SPMs with pointwise t-statistics and p-values (n = 17)

One part of the push phase (11–26%) that differed significantly between overground and treadmill propulsion (p = 0.012) was identified by the SPM (Fig. 4) for total force (F_tot_). Moreover, a part of the push phase (0–8%) was identified that differed between overground and ergometer propulsion. The SPM also identified one part of the push phase (6–29%) that differed significantly between overground and treadmill propulsion (p < 0.001) for torque (Fig. 5). Additionally, a part of the push phase (10–22%) was found to differ significantly between treadmill and ergometer propulsion.

## Discussion

The aim of the current study was to compare the physiological and biomechanical characteristics of handrim wheelchair propulsion in able-bodied participants in overground, treadmill, and ergometer conditions at similar submaximal power output, using conventional standardization methods. In general, almost all physiological, spatiotemporal, and kinetic variables showed moderate to good agreement among modalities without significant differences in level of agreement at the group level. About half of the variables were significantly different between conditions.

This was the first study that tried to actively standardize power output and speed between modalities. While no significant difference in power output was found, there was some within-subject variance as could be observed from the poor-moderate intraclass correlations and their large confidence intervals. Given the low mean power output, the absolute differences (up to 0.8 W) are small [4], but the relative differences (up to 17%) are rather large. Standardizing power output on the treadmill and ergometer at these low power outputs is almost impossible using current techniques [26] as small shifts in the, already dynamic, weight distribution will affect the rolling resistance and power output as a result [39]. The difference in speed was small and only present for the ergometer. It could be that the actual radius of the wheel/tyre was slightly lower in the ergometer condition due to the high pressure on the small tyre surface, leading to a slight mismatch between measured velocity by the measurement wheel and displayed velocity by the ergometer.

Heart rate was significantly higher during overground propulsion than during treadmill propulsion. While heart rate is more sensitive to emotional and social environmental distractions of the ‘open field’ [40], the overground modality also contained corners which require more effort to traverse than straight-line propulsion. Although the corners and short-straights were excluded from the data, the increased effort might have been reflected in the heart rate as it does not change instantaneously. This corresponds with the (non-significant) higher energy expenditure and somewhat lower mechanical efficiency that were observed when compared to treadmill and ergometer propulsion.

Push time was significantly different between conditions, but only for the ergometer. Contact angle was also different between overground and ergometer, and treadmill and ergometer propulsion. It is reasonable to expect both push time and contact angle to be highest on the ergometer as there is a reduced emphasis on steering compared to overground and treadmill propulsion [6, 23]. However, in this particular case, the real-time feedback of velocity as two numbers on a screen could have motivated participants to make fast adjustments to their speed to keep on target. Accordingly, future studies should look into the effect of different forms of speed/directional feedback on wheelchair ergometers. The somewhat larger cycle time (and cadence) of overground versus treadmill/ergometer propulsion is in line with earlier studies on gait [11] and wheelchair propulsion [23]. It was expected that the relatively large familiarization time would reduce any possible differences. Cycle time is known to increase after practice on a treadmill [30, 41] and it was shown that familiarization reduces differences between modalities in gait [20].

In general, the kinetics obtained from all modalities were highly similar. Fraction of effective force was higher on the ergometer compared to the other two modalities which is in line with the reduced emphasis on steering and balancing. No significant differences and good agreement were found for discrete force related parameters (mean and maximum). However, the continuous analysis showed a different build-up of force/torque for the treadmill condition, highlighting the importance of a more detailed continuous analysis [28]. This difference could be attributed to a more cautious pushing approach as treadmill propulsion requires the most finesse due to its stringent steering requirements, though differences appear to be very small and should not be overstated.

A limitation in the study was that, despite standardizing power output between modalities, within-subject variance in power output was still present. However, as the conditions were not significantly different from each other at the group level, the approach did appear to work to an extent. Additionally, the feedback provided to participants of their speed was specific for the different conditions, which could have influenced participant behaviour. Adding similar task constraints to treadmill/ergometer as to overground propulsion in the form of Virtual Reality could provide a higher degree of standardization. Overground and ergometer propulsion will, however, still have different task requirements than treadmill propulsion as the latter does not generally allow for self-selected speeds. Furthermore, the study did not look at the kinematics among modalities. It could very well be that similar outcomes are reached with different kinematic solutions. Moreover, involvement of the trunk was attributed to differences found between treadmills and ergometers in the past [42]. Indeed, some of the differences found between ergometer and overground/treadmill propulsion could be explained by the involvement of the trunk as rotational stability is higher on an ergometer.

Finally, changes that have been reported for walking have been small and the differences that reached statistical significance may not be functionally meaningful [14]. Most of the differences reported here are below the smallest detectable differences reported in [4], indicating that this also could be the case for wheelchair propulsion. It should also be noted that the results in the current study were obtained in able-bodied participants that form a more homogenous group but are also less experienced in wheelchair propulsion [30]. Possible effects of experience and impairment remain unexplored. As such, future studies should look into the clinical relevance of these differences and their effects on specific measurement protocols used in (clinical) practice within patient populations.

## Conclusions

In general, overground, treadmill, and ergometer wheelchair propulsion provide quite similar physiological and biomechanical outcomes after standardizing power output, speed, and a familiarization period. Some small differences were found in a number of physiological, spatiotemporal, and kinetic parameters. Moreover, the build-up of the push phase is different between modalities. However, differences are small and unlikely to be functionally meaningful. Future studies should look into the possible sources of differences and the clinical relevance of differences between modalities by comparing testing protocols. Under the constraints of identical standardized power output and velocity, the studied modalities of handrim wheelchair propulsion appear quite comparable.

## Data Availability

The datasets used and/or analysed during the current study are available from the corresponding author on reasonable request.

## Abbreviations

ICC: Intraclass correlations
OG: Overground
RM-ANOVA: Repeated-measures analysis of variance
SPM: Statistical parametric mapping
TM: Treadmill
WE: Wheelchair ergometer
